# The Mutagenic Consequences of DNA Methylation within and across Generations

**DOI:** 10.3390/epigenomes6040033

**Published:** 2022-10-04

**Authors:** Haley E. Hanson, Andrea L. Liebl

**Affiliations:** 1 Global and Planetary Health, University of South Florida, Tampa, FL 33620, USA; 2 Department of Biology, University of South Dakota, Vermillion, SD 57069, USA

**Keywords:** environmental epigenetics, DNA methylation, mutation, evolution

## Abstract

DNA methylation is an epigenetic modification with wide-ranging consequences across the life of an organism. This modification can be stable, persisting through development despite changing environmental conditions. However, in other contexts, DNA methylation can also be flexible, underlying organismal phenotypic plasticity. One underappreciated aspect of DNA methylation is that it is a potent mutagen; methylated cytosines mutate at a much faster rate than other genetic motifs. This mutagenic property of DNA methylation has been largely ignored in eco-evolutionary literature, despite its prevalence. Here, we explore how DNA methylation induced by environmental and other factors could promote mutation and lead to evolutionary change at a more rapid rate and in a more directed manner than through stochastic genetic mutations alone. We argue for future research on the evolutionary implications of DNA methylation driven mutations both within the lifetime of organisms, as well as across timescales.

## 1. Introduction

**Figure 1:**
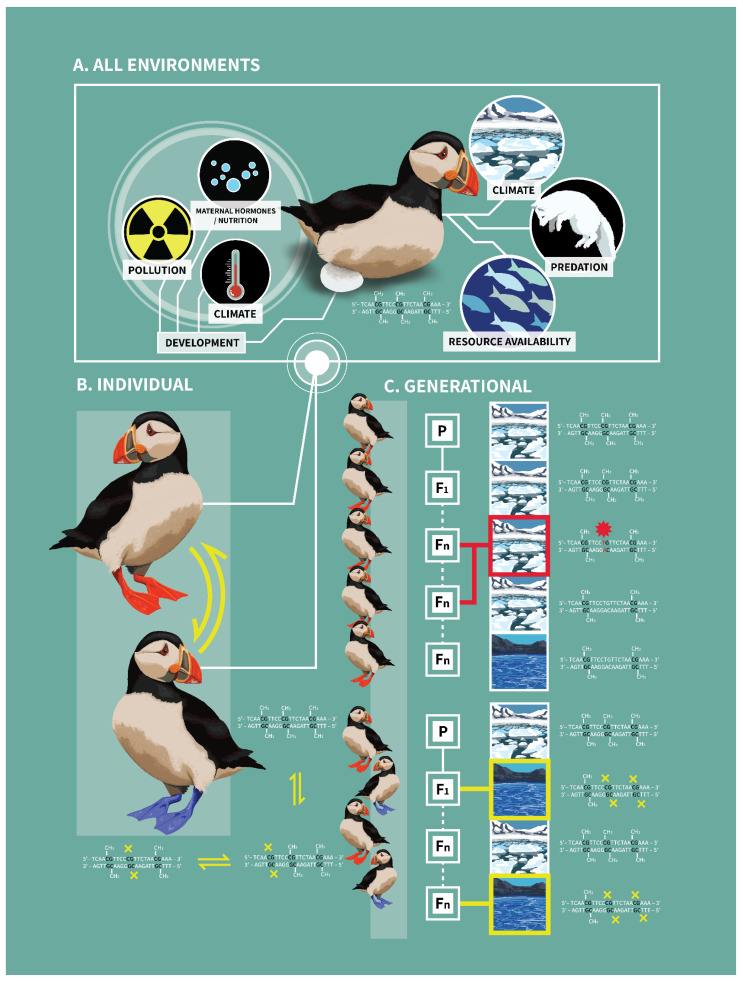
**Connecting DNA methylation within individual and across generations.** The environmental factors that shape DNA methylation patterns in individuals are numerous and occur throughout the lifetime of an individual: these vary from maternal hormones derived during development to predation events and resource availability (Panel (**A**)). Further, exposure to environmental stimuli can lead to variable methylation patterns at particular CpG sites through life, which can lead to variable phenotypes (denoted by two-directional arrows in Panel (**B**)). If environmental stimuli and resulting methylation are stable across generations (Panel (**C**), top), the CpG site is more likely to become methylated and mutate over time, possibly fixing that phenotype; if, on the other hand, environmental stimuli vary between generations (Panel (**C**), bottom), methylation is more likely to continue to regulate the phenotype at that CpG site. Here, we use a fictitious example in which puffins are exposed to either variable temperatures (Panel (**C**), bottom) or stable temperatures (Panel (**C**), top). When the environment is cold, more CpG sites are methylated and high levels of methylation confers the orange feet phenotype, an advantageous trait in cold environments. When the environment warms, fewer CpG sites are methylated and foot color becomes blue, an advantageous trait in warmer environments. If temperature persistence across generations leads to predictable methylation of CpG sites, this could lead to CpG to TpG or CpA mutations (denoted by a red star in Panel (**C**), top). These mutations may fix the orange foot phenotype, even in the absence of the colder temperatures (i.e., genetic assimilation); this represents the loss of epigenetic potential, leading to less flexibility in phenotype. In cold environments, this fixation of the phenotype could be advantageous if offspring are “primed” for predicted environmental conditions. However, if the environment changes and becomes warmer over time, this once advantageous mutation might become deleterious. If there is exposure to a variety of different environmental cues between generations, CpG sites may not be lost as methylation was variable (denoted by yellow boxes in Panel (**C**), bottom). Consequently, epigenetic potential is maintained, and the capacity for phenotypic plasticity is preserved. Note: the figure and phenotypes (e.g., foot color) represent a fictional example; there is no evidence that foot color is derived via epigenetic mechanisms, as depicted. This figure was created by Katie Brust.

DNA methylation is an epigenetic modification that influences the regulation of transcriptional activity and gene expression, leading to changes in phenotype. It is both persistent across rounds of cellular replication as well as responsive to endogenous and environmental cues to both stably and flexibly shape phenotypes [1] (Figure 1A). It is ubiquitous and fundamental to survival, required for many developmental processes, and essential for flexibility in many physiological, behavioral, and morphological traits [2]. However, this mechanism that helps shape phenotypes has an additional evolutionary consequence: it can act as a potent mutagen, having the potential to alter the phenotype outright as well as constrain future DNA methylation at that site [3,4]. DNA methylation is observed across all three domains of life: bacteria, archaea, and eukarya, as well as in many viruses [5,6,7]. The mechanisms by which methylation is added and removed throughout the genome has changed through evolutionary time, leading to differences in the location, frequency, and function of DNA methylation across taxa [7,8]; as a result, the effect of methylation on mutations has also diverged across evolution. Here, focusing on vertebrate methylation as an example, we synthesize literature on DNA methylation and assess how it may impact organisms within and across generations- through changes in methylation status itself as well as how it may contribute to molecular evolution. First, we describe the structure, machinery, and mechanisms governing DNA methylation, the pathways by which it can impact phenotype, and how it leads to variation in traits within and among individuals. We then explore the implications of DNA methylation both within and across generations. Finally, we consider the fundamental nature of DNA methylation as a mutagen and how methylation driven mutations may promote rapid evolutionary change.

## 2. DNA Methylation: Structure, Machinery, Mechanism, and Regulation

There are tens of thousands of protein coding genes in the vertebrate genome which must be activated and suppressed specifically and accurately across cells, tissues, time, and context [9,10]; DNA methylation and demethylation help regulate such activation and suppression. In vertebrate genomes, methylation most typically occurs at CpG sites, which confer both genetic and epigenetic information: CpG sites are not only encoded in the DNA sequence but are also interpreted for the presence or absence of methylation [11,12]. CpG sites are commonly found in gene promotors, intergenic regions of genes, transposable elements, and repetitive motifs. CpG sites near transcription start sites in gene promoters are generally hypomethylated across vertebrate taxa [13,14], whereas CpG sites in intragenic regions of actively transcribed genes, transposable elements, and repetitive sequences are generally methylated [1,15,16,17,18,19,20]. DNA methylation at a CpG island in the promoter region of a gene can influence the affinity for transcription factor binding [21]: although some transcription factors are inhibited completely by the presence of methyl groups [13,22], others preferentially bind to methylated binding sites [23] or some can only bind when DNA methylation is present [24,25]. Further, in the gene body itself, methylation can regulate alternative promoters [26], facilitate alternative splicing [27], enhance transcription, or suppress transposable element expression [13]; transposable elements harbor a significant portion of the genome’s CpG sites and often exhibit high levels of methylation to inhibit their expression [15]. Interestingly, regions of high CpG density are correlated with low levels of methylation that is highly conserved; conversely, CpG islands in more intermediate and low densities tend to be more variably methylated across tissues and taxa [14,16,28]. The number of CpG sites in a gene or genome represents its epigenetic potential, or the latent capacity for DNA methylation to occur and contribute to phenotypic plasticity [29]. Each CpG site represents a place where methylation, and consequently gene regulation, can occur.

DNA methylation is catalyzed by a suite of enzymes, most notably the DNA methyltransferases (DNMTs), which transfer a methyl group from *S*-adenosyl methionine (SAM) to the 5′ position of a pyrimidine ring of cytosines in a CpG site. With de novo methylation, methyl groups are added most frequently by DNMT3A and DNMT3B [30]. DNMT3A methylates imprinted genes and repeated elements, whereas DNMT3B methylates actively transcribed genes in the gene body [31,32]. Both enzymes also work in concert with DNMT3L [1,33,34]. DNMT1 can also catalyze de novo methylation under certain circumstances [30,35,36], but it works primarily to add methylation to hemi-methylated DNA (whereby methylation is added to a newly replicated strand of DNA to maintain methylation through DNA replication and cell division) [37]; preservation of the cellular state through time is dependent on the precise copying of these marks [23,38]. Demethylation occurs both passively and actively. Passively, DNA methylation can be depleted if DNMT1 activity is reduced or absent, consequently constraining the copying of methylation across rounds of DNA replication and cell division [39,40,41]. Active demethylation, on the other hand, occurs via the oxidation of methylated cytosine to 5-hydroxymethylcytosine, 5-formylcytosine, and 5-carboxyylctyosine by ten eleven translocation (TET) enzymes [42]. Oxidized cytosines are removed by thymine DNA glycosylase, and an unmodified cytosine is then restored through base excision repair [40]. Other active mechanisms of DNA methylation removal have also been proposed (e.g., via AID/APOBEC and DNA demethylase enzymes) but their contribution is contested [43,44,45].

One fundamental question remains: How are specific CpG sites targeted for methylation and demethylation? We know that the tendency of individual CpG sites to be methylated is non-random and regulated. For instance, the closer two CpG sites are to one another, the more likely they are to share methylation status [46]. However, the specific mechanisms that methylate (or demethylate) some CpG sites but not others are complex and include several non-mutually exclusive pathways that have not yet fully been elucidated. DNMT3A is likely able to target CpG islands through its PWWP domain [47]. Another way that specific CpG sites are targeted for methylation or demethylation is through the binding of transcription factors and other proteins (Reviewed in [23,48]). Further, DNMTs interact with and are regulated by other proteins, enzymes, and RNA, and there are numerous proteins that can recruit DNMTs to methylate localized CpG sites [49,50]. Additionally, some RNA, such as long non-coding RNA, can bind to DNMTs to suppress or inhibit their activity [50,51,52]. Specifically, PIWI-interacting RNA (piRNA) in the germline can induce DNA methylation at specific target sites that silence transposable elements (Reviewed in [53]). piRNAs are also found in the soma, but their function there is more obscure [54]. Similar processes likely guide the active demethylation process [55], but these processes are not as clear. A better understanding of how methylation is added and removed from specific CpG sites will help us to better understand not only the mechanisms by which methylation can control phenotypes, but also how methylation may influence and be influenced by evolution.

## 3. Implications of DNA Methylation within and across Generations

Within an individual, DNA methylation can act both as a flexible response to environmental cues, changing in response to changes in the internal and external environment, as well as allowing for persistent phenotypes in which stable marks lead to differences between cells, tissues, individuals, and populations [56,57,58]; stable marks can either be induced by developmental environments or inherited from parents. DNA methylation is responsive to a broad range of environmental factors, including but not limited to, parental care [59,60], temperature [61], salinity [62], resource availability [63], resource defense [64], social environment [65], climate [66], and pollution (Figure 1A) [67]. Both flexible and stable DNA methylation have phenotypic consequences. As the effects of DNA methylation on phenotypes have been reviewed and is not the focus on the current manuscript, the discussion below is not exhaustive [68,69,70,71].

Some methyl marks can be induced and removed in response to endogenous cues and environmental factors, both accustomed and unfamiliar, and are reversible (Figure 1A). Remarkably, such DNA methylation has the capacity to change rapidly, with many studies citing changes in DNA methylation or demethylation of specific CpG sites within just minutes of a stimulus [64,72,73,74,75]. For instance, in one study, mice were injected with an endotoxin to instigate an immune response. The endotoxin injection led to the activation of Interleukin-2 (*IL-2*), responsible for the proliferation of T cells, via demethylation of specific CpG sites in the promoter region of the gene within 20 min of endotoxin administration [76]. The ability of DNA methylation to regulate gene expression rapidly permits a temporary response to a stimulus, to cope and return to homeostasis. The rate of such alterations is likely one way individuals survive rapid environmental changes [77]. Thus, DNA methylation is one mechanism underpinning phenotypic plasticity, or the capability for one genotype to produce multiple phenotypes in response to environmental variation [78,79]. Some DNA methylation marks change through time predictably in response to environmental cycles, such as circadian and seasonal changes [80,81,82,83,84,85]. For example, in Siberian hamsters (*Phodopus sungorus*), photoperiod drives changes to reproductive physiology, with short photoperiods in winter months leading to gonadal regression and longer photoperiods in the summer reversing this [80]. These changes are facilitated by DNA methylation and demethylation of the promoter of the deiodinase type III (*dio3*) gene, a key regulator of thyroid hormone, such that winter photoperiods reduced and summer photo periods induced DNA methylation [80]. However, unexpected environmental perturbations can also induce reversible DNA methylation through time. Although within individual, repeated measure studies are relatively rare in the ecological epigenetics literature, inducible changes to DNA methylation have been shown in response to changes in diet and temperature, among other environmental variables [61,62,63,86]. In humans, examples of reversible methylation marks also exist (e.g., [87,88,89]).

Some DNA methylation induced from the environment (usually during development) may become a stable mark, maintained through DNA replication and cell division throughout an individual’s life. Phenotypic differences in clonal populations or monozygotic twins can be explained by differential patterns of DNA methylation induced early in life [90,91]. Perhaps one of the most dramatic developmentally derived phenotypes is temperature dependent sex determination in some fish and reptiles. Research across several species indicates temperature determines sex via differential methylation patterns in the promoters of the *cyp19a* and *SOX9* genes [92,93,94]. Other developmental environments have also been shown to have lasting phenotypic effects through impacts on methylation such as parental care, rainfall, and nutritional environment [60,65,66,95].

One of the most contested topics concerning DNA methylation, and epigenetic modifications in general, is its capacity to be transmitted across generations (Figure 1C) [96]. Epigenetic inheritance is thought to be advantageous as it could prime offspring for environmental conditions they are likely to face [97,98]. Although epigenetic signals are known to at least sometimes be stable and heritable, it has largely been studied in plants (for a review see [99]) and had long been dismissed in vertebrates due to extensive demethylation during embryogenesis and in primordial germ cells [97,100,101,102,103]. However, evidence now exists that this phenomenon occurs in vertebrates [97,98,104,105,106]. It is now understood that not all DNA methylation is removed during embryogenesis, and other mechanisms may recapitulate marks faithfully [98,101]. Several recent studies in three-spined stickleback (*Gasterosteus aculeatus*) indicates that a large proportion of methylation could be heritable and stable across generations [107,108]. Given its importance of passing information across generations it is unsurprising that evidence from theoretical models suggests that epigenetic inheritance would impact the both rate and trajectory of evolutionary change (Reviewed in [77]).

## 4. Mutation of CpG Sites

CpG sites mutate 10-50 times faster than any other genomic motif [109,110]. This mutability is attributable to DNA methylation: a methylated cytosine is an order of magnitude more likely to mutate than an unmethylated cytosine [4,111]. Obviously, not every CpG site mutation is a direct result of methylation and some mutations will occur by chance at unmodified CpG sites. However, this fraction will be extremely small, as the mutation rate of methylated cytosines is nearly 20,000 times that of unmethylated cytosines [96,112]. In addition to somatic cells (Figure 1A), CpG mutations are also expected to occur in the germline (Figure 1C), which will influence how such mutations are inherited [113]. Sperm cells, for instance, are highly methylated compared to other cell types and CpG mutations correlate with male age in humans [114]. As these mutations accumulate over time, other life-history traits, including generation time may also affect the number of mutations passed on [18,115].

CpG sites are mutated through spontaneous hydrolytic deamination. Whereas cytosine deamination yields uracil, deamination of methylated cytosine generates thymine [4]. Uracil, a foreign base in DNA, is identified and removed by uracil-DNA glycosylase and replaced with a cytosine, leaving the CpG site intact [4]. Conversely, the thymine mutation is less efficiently recognized [116]. Two enzymes, thymine DNA glycosylase and methyl-binding domain protein 4, can recognize the foreign thymine, but how they specifically target deamination products and what regulates their efficiency remains unresolved (Box 1) [117,118,119]. If the thymine is not replaced before replication, the mutation persists, resulting in a CpG to TpG or CpA mutation, depending on the orientation of the methylated cytosine [117]. Recent studies suggest that other factors, including surrounding sequence variation, may further contribute to the rate of CpG mutation via deamination [120,121,122,123,124]. As such, TpG and CpA transitions are the most common type of mutation in vertebrate genomes [125]. Unlike other de novo mutations, the mutation of methylated cytosines is independent of cell divisions or replication, and instead accumulate in a time-dependent manner [110,126,127]. Indeed, the accumulation of CpG to TpG or CpA mutations increases with age in humans, the only mutational signature to do so [128,129,130]. Together, this suggests that the longer a CpG site is methylated, the more likely it is to undergo deamination to thymine [131]. In other words, methylation-dependent mutations are more likely to occur at sites that are stably methylated through time. Therefore, when environments dictate methylation status and those environments remain stable through time, resulting in a stable methyl mark, those sites are more likely to mutate. This will reduce the ability of methylation to regulate the phenotype, thus conferring a more stable (albeit not necessarily advantageous) phenotype that is subject to evolution. Conversely, if changes in the environment result in a CpG site only sometimes being methylated, then mutations are less likely to occur (Figure 1C). In addition, new CpG sites can also be created. Stochastic mutations can occur leading the creation of a new CpG site, however, the most frequently invoked mechanism of CpG site gain is through GC-biased gene conversion [12].

Box 1 **Outstanding Questions.**

What is the timescale over which DNA methylation attributed mutations occur? In other words, how many generations might methylation need to persist to lead to mutation?

Do some environmental contexts (e.g., unpredictable environments, range expansions) delay or accelerate CpG mutation?

What factors that contribute to the efficacy and specificity of deamination repair (e.g., TGD and MBD4)? How much individual variation exists in these pathways?

What is the impact of these mutations relative to other evolutionary mechanisms, such as stochastic de novo mutations, on the rate and direction of evolutionary change?

Do DNA methylation driven mutations cause neutral, deleterious, or adaptive effects at the same rate as stochastic mutations? Are these changes more or less reliant on the environment than stochastic mutations are?

Much like DNA methylation, the loss of CpG sites does not occur at a uniform rate across the genome. Regions of dense CpG sites show reduced rates of CpG depletion compared to other regions [132,133]. This may occur due to two non-mutually exclusive mechanisms: hypomethylation of these regions in the germline may buffer them from mutation, or selection may be acting to preserve CpG sites in these regions [113,134]. For instance, CpG sites in protein-coding regions of genes vital for development (e.g., *Hox* genes) are strongly selected for [125]. Conversely, regions of low to intermediate levels of methylation, especially within intergenic or intronic regions, generally have increased rates of CpG mutation [135]. Thus, in genes where there are functional constrains or where tight gene regulation is necessary, CpG sites are likely more buffered from deamination, whereas in genes in which plasticity or regulatory flexibility is common, mutation may occur more frequently.

Because of the mutability of CpG sites when methylation is present, DNA methylation has an exceedingly complex, yet more important role in evolution than has been previously appreciated. CpG mutations effectively remove the substrate on which DNA methylation can act, leading to a permanent reduction in the capacity for DNA methylation-based gene regulation [3,136,137]. The loss of methylation can then impact how and when genes are expressed. Where the CpG site is located in the genome will further influence its effect [138,139]. CpG mutations can also underlie allele-specific methylation, and subsequently allele-specific expression [3,140] as well as other aspects of gene regulation, such as transcription factor binding sites [141].

## 5. Epigenetic Potential

The number of CpG sites represents a form of epigenetic potential, or the latent capacity for DNA methylation to occur and contribute to phenotypic plasticity [29]. Each CpG site represents a place in the genome where methylation, and consequently gene regulation might occur. The depletion of CpG sites represents the winnowing of epigenetic potential as lost CpG site can no longer mediate gene regulation (Figure 1B) [29,142]. Therefore, the number of CpG sites represent a genetic constraint, and CpG depletion likely has many impacts on fitness [143]. For instance, high levels of phenotypic plasticity might be favored under certain conditions, such as in invasive species or naturally expanding populations coping with novel environmental conditions [144,145]. In these situations, depletion of CpG sites may impede the ability for individuals, populations, or species to cope with novel environments; thus, CpG sites may be selected to prevent the erosion of plasticity. One of the most successful invasive species, the house sparrow (*Passer domesticus*), shows differences in epigenetic regulation throughout their native and invasive ranges [146,147]. Further, across 70 years of one of their most recent introductions (to Kenya), house sparrow populations show differences in the number of their CpG sites, and thus their epigenetic potential [137]. Specifically, birds at the range edge (established for ~5 generations), maintained significantly more CpG sites than their conspecifics found at the site of the introduction (established for ~50 generations). These trends seem to have emerged through selection on CpG sites in birds at the range edge, suggesting that changes in CpG sites occurred across a relatively rapid time scale. Importantly, these changes in epigenetic potential are concurrent with expected changes in plasticity and the ability to cope with novel environments [146,148,149,150].

## 6. The Evolutionary Consequences of CpG Mutations

CpG mutations induced by methylation certainly contribute to adaptive evolution [151]. As methylated cytosines mutate faster than any other genomic motif [152,153,154], DNA methylation alters the rate and direction of evolution by acting as a mechanism to increase genetic diversity that can lead to changes in genetic regulation more quickly than stochastic mutations. CpG mutations can lead to adaptive changes over multiple evolutionary timescales. Over shorter evolutionary timescales, the mutations caused by DNA methylation may be a mechanism leading to genetic assimilation, or the process by which a phenotype produced in response to a stimulus becomes genetically encoded (Figure 1C) [96,155,156]. Through mechanisms described above, environmental stimuli can induce DNA methylation at specific CpG sites that produce adaptive changes in a phenotype. If the stimulus persists over multiple generations, methylation and the altered phenotype should also persist. However, although methylation continues to induce the altered phenotype, it also increases the likelihood of mutation at that specific CpG site as CpG sites are more likely to become mutated the longer they are methylated [131]. Once mutated, although DNA methylation would no longer be able to regulate the phenotype, in some cases, the phenotype may become fixed with the CpG mutation. This change would be adaptive if the time the CpG site is methylated acts as a signal that environmental conditions are consistent over several generations [157]. This ‘inheritance relay’ from the flexible phenotypic induction of DNA methylation to one from a more persistent genetic change would likely greatly accelerate genetic adaptation through genetic assimilation [96].

In addition to genetic assimilation, the greatly increased mutation rate associated with CpG methylation has several effects on the genome, and therefore on evolution. In vertebrates, CpG sites are found five-fold less frequently than expected [158,159], largely due to the impact DNA methylation has on these sites [157]. CpG depletion influences the number of transcription factor binding sites found throughout the genome, which, in turn, influences how and when genes are expressed. For example, transposable elements, which have a high density of CpG sites, experience high rates of deamination and mutation [160]. These mutations to CpG sites in transposable elements are responsible for the creation of transcription factor binding sites, and are hypothesized to have created new opportunities for gene regulation that are often specific to a species [15,160,161,162]. Further, methylation driven CpG mutation can also create new transcription factor binding sites in promoter region of genes [163].

One particularly salient example of an adaptive change caused by CpG mutation is from a study of free-living Andean house wrens (*Troglodytes aedon*) by Galen et al. (2015). In Peru, Andean house wrens inhabit a range of altitudes, from sea level to above 4500 m. At different altitudes, they exhibit changes in hemoglobin’s oxygen affinity such that birds living at the highest altitudes have the highest levels of oxygen affinity, which is enhanced by more than 30% compared to birds living at lower altitudes. This change is attributable to an ancestral CpG site mutating to a CpA, leading to a nonsynonymous substitution. The frequency of the mutated allele linearly tracks with altitude, such that populations living at high altitudes have fixed the mutated CpA site, whereas low altitude populations have negligible levels of the mutation with all individuals retaining the CpG site [164]. Further, when comparing hemoglobin’s oxygen affinity in 35 pairs of high and low altitude avian species, increased oxygen affinity of hemoglobin only occurs in the high altitude species and CpG mutations are the overrepresented cause of those changes [151].

CpG mutations also play a major role in divergence and speciation [165]. Across the genome, humans and chimpanzees differ by less than 1%; however, when considering variation only at CpG sites, divergence between the two species increases to over 15% [12,166]. This difference is caused by losses and gains of CpG motifs at an approximately equal rate; nevertheless, the stark contrast in the magnitude of divergence between the whole genome and CpG sites alone highlights the importance of these sites for evolution [166]. In addition, CpG mutations have contributed to diversification amongst domesticated chicken and their closest living relative, the Red Jungle Fowl (*Gallus gallus*) [167]. As genetic divergence has increased from the Red Jungle Fowl, so too has the number of CpG mutations, which are significantly overrepresented compared to other mutation types [167].

As with stochastic mutations, not all, or even most, of the mutations generated by DNA methylation are likely to be adaptive. In fact, methylation driven CpG mutations are best studied in the context of human disease [168]. In one such study utilizing the Human Gene Mutation Database, CpG mutations were identified as the cause of 18.2% of all mutations causing inherited diseases, more than 10 times the expected rate [169]. Further, within somatic cells, mutations driven by methylation is frequently detected across multiple types of cancer [170]. Although the focus of this review is on evolutionary consequences of DNA methylation mutations, they have, unfortunately, been less well studied than the human disease implications of CpG mutation. We argue that one key area of future research should focus on the consequences of mutations that result from DNA methylation in ecological settings (Box 1). Unraveling this mystery will likely have significant ramifications for understanding how methylation driven mutations contribute to evolution in natural environments.

## 7. Concluding Remarks

Unequivocally, DNA methylation is vital for the survival of all organisms: methylation contributes to the development of complex phenotypes and is important for many facets of transcriptional regulation [2]. Importantly, methylation is not inherently permanent. Many intrinsic or environmental stimuli can impact methylation, leading to changes in phenotype. Despite this, however, here we argue that methylation also impacts the evolution of vertebrate genomes more than previously appreciated, namely through its influence on mutation of CpG sites. Because of the impact of methylation, CpG mutations are not entirely random. As methylation predates mutations, it seems highly unlikely that environmental conditions cannot and have not shaped patterns of mutation; however, whether and how environmental cues lead to the methylation and subsequent mutation of *specific* CpG sites is currently unknown (Box 1). It is possible that the semi-directed nature in which specific CpG sites are methylated in response to environmental cues initiates the switch to adaptive phenotypes. However, as with stochastic mutations, not all CpG mutations will be advantageous. For these reasons, it is vital that we investigate DNA methylation and the attributed mutations with an emphasis on intermediate timescales, where mutations leading to genetic assimilation might be particularly evident, to better understand whether and how this phenomenon occurs over relatively rapid periods of time and what effects this may have on the rate and direction of evolution (Box 1).

## Data Availability

Not applicable.

## References

[1] Greenberg M.V.C., Bourc’his D. (2019). The diverse roles of DNA methylation in mammalian development and disease. Nat. Rev. Mol. Cell Biol..

[2] Smith Z.D., Meissner A. (2013). DNA methylation: Roles in mammalian development. Nat. Rev. Genet..

[3] Zhou D., Li Z., Yu D., Wan L., Zhu Y., Lai M., Zhang D. (2015). Polymorphisms involving gain or loss of CpG sites are significantly enriched in trait-associated SNPs. Oncotarget.

[4] Nabel C.S., Manning S.A., Kohli R.M. (2012). The curious chemical biology of cytosine: Deamination, methylation, and oxidation as modulators of genomic potential. ACS Chem. Biol..

[5] Blow M.J., Clark T.A., Daum C.G., Deutschbauer A.M., Fomenkov A., Fries R., Froula J., Kang D.D., Malmstrom R.R., Morgan R.D. (2016). The Epigenomic Landscape of Prokaryotes. PLoS Genet..

[6] Hoelzer K., Shackelton L.A., Parrish C.R. (2008). Presence and role of cytosine methylation in DNA viruses of animals. Nucleic Acids Res..

[7] Iyer L.M., Abhiman S., Aravind L. (2011). Natural History of Eukaryotic DNA Methylation Systems.

[8] Ponger L., Li W.H. (2005). Evolutionary diversification of DNA methyltransferases in eukaryotic genomes. Mol. Biol. Evol..

[9] Reik W. (2007). Stability and flexibility of epigenetic gene regulation in mammalian development. Nature.

[10] Ficz G. (2015). New insights into mechanisms that regulate DNA methylation patterning. J. Exp. Biol..

[11] Lister R., Pelizzola M., Dowen R., Hawkins R., Hon G., Nery J., Lee L., Ye Z., Ngo Q., Edsall L. (2009). Human DNA methylomes at base resolution show widespread epigenomic differences. Nature.

[12] Bell C.G., Wilson G.A., Butcher L.M., Roos C., Walter L., Beck S. (2012). Human-specific CpG “beacons” identify loci associated with human-specific traits and disease. Epigenetics.

[13] Jones P.A. (2012). Functions of DNA methylation: Islands, start sites, gene bodies and beyond. Nat. Rev. Genet..

[14] Long H.K., Sims D., Heger A., Blackledge N.P., Kutter C., Wright M.L., Grützner F., Odom D.T., Patient R., Ponting C.P. (2013). Epigenetic conservation at gene regulatory elements revealed by non-methylated DNA profiling in seven vertebrates. Elife.

[15] Xiang S., Liu Z., Zhang B., Zhou J., Zhu B.D., Ji J., Deng D. (2010). Methylation status of individual CpG sites within Alu elements in the human genome and Alu hypomethylation in gastric carcinomas. BMC Cancer.

[16] Xin Y., O’Donnell A.H., Ge Y., Chanrion B., Milekic M., Rosoklija G., Stankov A., Arango V., Dwork A.J., Gingrich J.A. (2011). Role of CpG context and content in evolutionary signatures of brain DNA methylation. Epigenetics.

[17] Zemach A., McDaniel I.E., Silva P., Zilberman D. (2010). Genome-wide evolutionary analysis of eukaryotic DNA methylation. Science.

[18] Varriale A. (2014). DNA Methylation, Epigenetics, and Evolution in Vertebrates: Facts and Challenges. Int. J. Evol. Biol..

[19] Suzuki M.M., Bird A. (2008). DNA methylation landscapes: Provocative insights from epigenomics. Nat. Rev. Genet..

[20] Feng S., Cokus S.J., Zhang X., Chen P.Y., Bostick M., Goll M.G., Hetzel J., Jain J., Strauss S.H., Halpern M.E. (2010). Conservation and divergence of methylation patterning in plants and animals. Proc. Natl. Acad. Sci. USA.

[21] Zhu H., Wang G., Qian J. (2016). Transcription factors as readers and effectors of DNA methylation. Nat. Rev. Genet..

[22] Tate P.H., Bird A.P. (1993). Effects of DNA methylation on DNA-binding proteins and gene expression. Curr. Opin. Genet. Dev..

[23] Yin Y., Morgunova E., Jolma A., Kaasinen E., Sahu B., Khund-Sayeed S., Das P.K., Kivioja T., Dave K., Zhong F. (2017). Impact of cytosine methylation on DNA binding specificities of human transcription factors. Science.

[24] Rishi V., Bhattacharya P., Chatterjee R., Rozenberg J., Zhao J., Glass K., Fitzgerald P., Vinson C. (2010). CpG methylation of half-CRE sequences creates C/EBPα binding sites that activate some tissue-specific genes. Proc. Natl. Acad. Sci. USA.

[25] Hu S., Wan J., Su Y., Song Q., Zeng Y., Nguyen H.N., Shin J., Cox E., Rho H.S., Woodard C. (2013). DNA methylation presents distinct binding sites for human transcription factors. Elife.

[26] Maunakea A.K., Nagarajan R.P., Bilenky M., Ballinger T.J., Dsouza C., Fouse S.D., Johnson B.E., Hong C., Nielsen C., Zhao Y. (2010). Conserved role of intragenic DNA methylation in regulating alternative promoters. Nature.

[27] Laurent L., Wong E., Li G., Huynh T., Tsirigos A., Ong C.T., Low H.M., Sung K.W.K., Rigoutsos I., Loring J. (2010). Dynamic changes in the human methylome during differentiation. Genome Res..

[28] Irizarry R.A., Ladd-Acosta C., Wen B., Wu Z., Montano C., Onyango P., Cui H., Gabo K., Rongione M., Webster M. (2009). The human colon cancer methylome shows similar hypo- and hypermethylation at conserved tissue-specific CpG island shores. Nat. Genet..

[29] Kilvitis H.J., Hanson H., Schrey A.W., Martin L.B. (2017). Epigenetic potential as a mechanism of phenotypic plasticity in vertebrate range expansions. Integr. Comp. Biol..

[30] Bestor T.H. (2000). The DNA methyltransferases of mammals. Hum. Mol. Genet..

[31] Baubec T., Colombo D.F., Wirbelauer C., Schmidt J., Burger L., Krebs A.R., Akalin A., Schübeler D. (2015). Genomic profiling of DNA methyltransferases reveals a role for DNMT3B in genic methylation. Nature.

[32] Neri F., Rapelli S., Krepelova A., Incarnato D., Parlato C., Basile G., Maldotti M., Anselmi F., Oliviero S. (2017). Intragenic DNA methylation prevents spurious transcription initiation. Nature.

[33] Bourc’his D., Xu G.L., Lin C.S., Bollman B., Bestor T.H. (2001). Dnmt3L and the establishment of maternal genomic imprints. Science.

[34] Aapola U., Shibuya K., Scott H.S., Ollila J., Vihinen M., Heino M., Shintani A., Kawasaki K., Minoshima S., Krohn K. (2000). Isolation and initial characterization of a novel zinc finger gene, DNMT3L, on 21q22.3, related to the cytosine-5-methyltransferase 3 gene family. Genomics.

[35] Yarychkivska O., Shahabuddin Z., Comfort N., Boulard M., Bestor T.H. (2018). BAH domains and a histone-like motif in DNA methyltransferase 1 (DNMT1) regulate de novo and maintenance methylation in vivo. J. Biol. Chem..

[36] Li J.-Y., Pu M.-T., Hirasawa R., Li B.-Z., Huang Y.-N., Zeng R., Jing N.-H., Chen T., Li E., Sasaki H. (2007). Synergistic Function of DNA Methyltransferases Dnmt3a and Dnmt3b in the Methylation of Oct4 and Nanog. Mol. Cell. Biol..

[37] Li E., Zhang Y. (2014). DNA Methylation in Mammals. Cold Spring Harb. Perspect. Biol..

[38] Sharif J., Muto M., Takebayashi S.I., Suetake I., Iwamatsu A., Endo T.A., Shinga J., Mizutani-Koseki Y., Toyoda T., Okamura K. (2007). The SRA protein Np95 mediates epigenetic inheritance by recruiting Dnmt1 to methylated DNA. Nature.

[39] Kagiwada S., Kurimoto K., Hirota T., Yamaji M., Saitou M. (2013). Replication-coupled passive DNA demethylation for the erasure of genome imprints in mice. EMBO J..

[40] Wu X., Zhang Y. (2017). TET-mediated active DNA demethylation: Mechanism, function and beyond. Nat. Rev. Genet..

[41] Saitou M., Kagiwada S., Kurimoto K. (2012). Epigenetic reprogramming in mouse pre-implantation development and primordial germ cells. Development.

[42] Wu H., Zhang Y. (2014). Reversing DNA methylation: Mechanisms, genomics, and biological functions. Cell.

[43] Guo J.U., Su Y., Zhong C., Ming G.L., Song H. (2011). Hydroxylation of 5-methylcytosine by TET1 promotes active DNA demethylation in the adult brain. Cell.

[44] Nabel C.S., Jia H., Ye Y., Shen L., Goldschmidt H.L., Stivers J.T., Zhang Y., Kohli R.M. (2012). AID/APOBEC deaminases disfavor modified cytosines implicated in DNA demethylation. Nat. Chem. Biol..

[45] Kohli R.M., Zhang Y. (2013). TET enzymes, TDG and the dynamics of DNA demethylation. Nature.

[46] Affinito O., Palumbo D., Fierro A., Cuomo M., De Riso G., Monticelli A., Miele G., Chiariotti L., Cocozza S. (2020). Nucleotide distance influences co-methylation between nearby CpG sites. Genomics.

[47] Weinberg D.N., Rosenbaum P., Chen X., Barrows D., Horth C., Marunde M.R., Popova I.K., Gillespie Z.B., Keogh M.C., Lu C. (2021). Two competing mechanisms of DNMT3A recruitment regulate the dynamics of de novo DNA methylation at PRC1-targeted CpG islands. Nat. Genet..

[48] Blattler A., Farnham P.J. (2013). Cross-talk between site-specific transcription factors and DNA methylation states. J. Biol. Chem..

[49] Ravichandran M., Jurkowska R.Z., Jurkowski T.P. (2018). Target specificity of mammalian DNA methylation and demethylation machinery. Org. Biomol. Chem..

[50] Laisné M., Gupta N., Kirsh O., Pradhan S., Defossez P.A. (2018). Mechanisms of DNA methyltransferase recruitment in mammals. Genes.

[51] Johnsson P., Ackley A., Vidarsdottir L., Lui W.O., Corcoran M., Grandér D., Morris K.V. (2013). A pseudogene long-noncoding-RNA network regulates PTEN transcription and translation in human cells. Nat. Struct. Mol. Biol..

[52] Stathopoulou A., Chhetri J.B., Ambrose J.C., Estève P.O., Ji L., Erdjument-Bromage H., Zhang G., Neubert T.A., Pradhan S., Herrero J. (2017). A novel requirement for DROSHA in maintenance of mammalian CG methylation. Nucleic Acids Res..

[53] Ozata D.M., Gainetdinov I., Zoch A., O’Carroll D., Zamore P.D. (2019). PIWI-interacting RNAs: Small RNAs with big functions. Nat. Rev. Genet..

[54] Ross R.J., Weiner M.M., Lin H. (2014). PIWI proteins and PIWI-interacting RNAs in the soma. Nature.

[55] Zhang H., Zhu J.K. (2012). Active DNA demethylation in plants and animals. Cold Spring Harb. Symp. Quant. Biol..

[56] Lanata C.M., Chung S.A., Criswell L.A. (2018). DNA methylation 101: What is important to know about DNA methylation and its role in SLE risk and disease heterogeneity. Lupus Sci. Med..

[57] Zhang B., Zhou Y., Lin N., Lowdon R.F., Hong C., Nagarajan R.P., Cheng J.B., Li D., Stevens M., Lee H.J. (2013). Functional DNA methylation differences between tissues, cell types, and across individuals discovered using the M&M algorithm. Genome Res..

[58] Schultz M.D., He Y., Whitaker J.W., Hariharan M., Mukamel E.A., Leung D., Rajagopal N., Nery J.R., Urich M.A., Chen H. (2015). Human body epigenome maps reveal noncanonical DNA methylation variation. Nature.

[59] Weaver I.C.G. (2005). Reversal of Maternal Programming of Stress Responses in Adult Offspring through Methyl Supplementation: Altering Epigenetic Marking Later in Life. J. Neurosci..

[60] Weaver I.C.G., Cervoni N., Champagne F.A., D’Alessio A.C., Sharma S., Seckl J.R., Dymov S., Szyf M., Meaney M.J. (2004). Epigenetic programming by maternal behavior. Nat. Neurosci..

[61] Metzger D.C.H., Schulte P.M. (2017). Persistent and plastic effects of temperature on dna methylation across the genome of threespine stickleback (*Gasterosteus aculeatus*). Proc. R. Soc. B Biol. Sci..

[62] Morán P., Marco-Rius F., Megías M., Covelo-Soto L., Pérez-Figueroa A. (2013). Environmental induced methylation changes associated with seawater adaptation in brown trout. Aquaculture.

[63] Lea A.J., Altmann J., Alberts S.C., Tung J. (2016). Resource base influences genome-wide DNA methylation levels in wild baboons (*Papio cynocephalus*). Mol. Ecol..

[64] Herb B.R., Shook M.S., Fields C.J., Robinson G.E. (2018). Defense against territorial intrusion is associated with DNA methylation changes in the honey bee brain. BMC Genom..

[65] McDade T.W., Ryan C., Jones M.J., MacIsaac J.L., Morin A.M., Meyer J.M., Borja J.B., Miller G.E., Kobor M.S., Kuzawa C.W. (2017). Social and physical environments early in development predict DNA methylation of inflammatory genes in young adulthood. Proc. Natl. Acad. Sci. USA.

[66] Rubenstein D.R., Skolnik H., Berrio A., Champagne F.A., Phelps S., Solomon J. (2016). Sex-specific fitness effects of unpredictable early life conditions are associated with DNA methylation in the avian glucocorticoid receptor. Mol. Ecol..

[67] Head J.A. (2014). Patterns of DNA methylation in animals: An ecotoxicological perspective. J. Environ. Law.

[68] Angers B., Castonguay E., Massicotte R. (2010). Environmentally induced phenotypes and DNA methylation: How to deal with unpredictable conditions until the next generation and after. Mol. Ecol..

[69] Hu J., Barrett R.D.H. (2017). Epigenetics in natural animal populations. J. Evol. Biol..

[70] Laine V.N., Sepers B., Lindner M., Gawehns F., Ruuskanen S., van Oers K. (2022). An ecologist’s guide for studying DNA methylation variation in wild vertebrates. Mol. Ecol. Resour..

[71] Vogt G. (2022). Paradigm shifts in animal epigenetics: Research on non-model species leads to new insights into dependencies, functions and inheritance of DNA methylation. BioEssays.

[72] Barrès R., Yan J., Egan B., Treebak J.T., Rasmussen M., Fritz T., Caidahl K., Krook A., O’Gorman D.J., Zierath J.R. (2012). Acute exercise remodels promoter methylation in human skeletal muscle. Cell Metab..

[73] Wiechmann T., Röh S., Sauer S., Czamara D., Arloth J., Ködel M., Beintner M., Knop L., Menke A., Binder E.B. (2019). Identification of dynamic glucocorticoid-induced methylation changes at the FKBP5 locus. Clin. Epigenetics.

[74] Métivier R., Gallais R., Tiffoche C., Le Péron C., Jurkowska R.Z., Carmouche R.P., Ibberson D., Barath P., Demay F., Reid G. (2008). Cyclical DNA methylation of a transcriptionally active promoter. Nature.

[75] Kangaspeska S., Stride B., Métivier R., Polycarpou-Schwarz M., Ibberson D., Carmouche R.P., Benes V., Gannon F., Reid G. (2008). Transient cyclical methylation of promoter DNA. Nature.

[76] Bruniquel D., Schwartz R.H. (2003). Selective, stable demethylation of the interleukin-2 gene enhances transcription by an active process. Nat. Immunol..

[77] Bonduriansky R., Crean A.J., Day T. (2012). The implications of nongenetic inheritance for evolution in changing environments. Evol. Appl..

[78] West-Eberhard M.J. (2003). Developmental Plasticity and Evolution.

[79] Pigliucci M. (2005). Evolution of phenotypic plasticity: Where are we going now?. Trends Ecol. Evol..

[80] Stevenson T.J., Prendergast B.J. (2013). Reversible DNA methylation regulates seasonal photoperiodic time measurement. Proc. Natl. Acad. Sci. USA.

[81] Oh G., Koncevičius K., Ebrahimi S., Carlucci M., Groot D.E., Nair A., Zhang A., Kriščiūnas A., Oh E.S., Labrie V. (2019). Circadian oscillations of cytosine modification in humans contribute to epigenetic variability, aging, and complex disease 06 Biological Sciences 0604 Genetics. Genome Biol..

[82] Oh G., Ebrahimi S., Carlucci M., Zhang A., Nair A., Groot D.E., Labrie V., Jia P., Oh E.S., Jeremian R.H. (2018). Cytosine modifications exhibit circadian oscillations that are involved in epigenetic diversity and aging. Nat. Commun..

[83] Biggar Y., Storey K.B. (2014). Global DNA modifications suppress transcription in brown adipose tissue during hibernation. Cryobiology.

[84] Alvarado S., Mak T., Liu S., Storey K.B., Szyf M. (2015). Dynamic changes in global and gene-specific DNA methylation during hibernation in adult thirteen-lined ground squirrels, Ictidomys tridecemlineatus. J. Exp. Biol..

[85] Viitaniemi H.M., Verhagen I., Visser M.E., Honkela A., Van Oers K., Husby A., Meyer M. (2019). Seasonal Variation in Genome-Wide DNA Methylation Patterns and the Onset of Seasonal Timing of Reproduction in Great Tits. Genome Biol. Evol..

[86] Marsh A.G., Pasqualone A.A. (2014). DNA methylation and temperature stress in an Antarctic polychaete, Spiophanes tcherniai. Front. Physiol..

[87] Tsaprouni L.G., Yang T.P., Bell J., Dick K.J., Kanoni S., Nisbet J., Viñuela A., Grundberg E., Nelson C.P., Meduri E. (2014). Cigarette smoking reduces DNA methylation levels at multiple genomic loci but the effect is partially reversible upon cessation. Epigenetics.

[88] Zhang S.C., Wang M.Y., Feng J.R., Chang Y., Ji S.R., Wu Y. (2020). Reversible promoter methylation determines fluctuating expression of acute phase proteins. Elife.

[89] Dugué P.A., Jung C.H., Joo J.E., Wang X., Wong E.M., Makalic E., Schmidt D.F., Baglietto L., Severi G., Southey M.C. (2020). Smoking and blood DNA methylation: An epigenome-wide association study and assessment of reversibility. Epigenetics.

[90] Massicotte R., Whitelaw E., Angers B. (2011). DNA methylation: A source of random variation in natural populations. Epigenetics.

[91] Souren N.Y., Gerdes L.A., Lutsik P., Gasparoni G., Beltrán E., Salhab A., Kümpfel T., Weichenhan D., Plass C., Hohlfeld R. (2019). DNA methylation signatures of monozygotic twins clinically discordant for multiple sclerosis. Nat. Commun..

[92] Navarro-Martín L., Viñas J., Ribas L., Díaz N., Gutiérrez A., Di Croce L., Piferrer F. (2011). DNA methylation of the gonadal aromatase (cyp19a) promoter is involved in temperature-dependent sex ratio shifts in the European sea bass. PLoS Genet..

[93] Matsumoto Y., Buemio A., Chu R., Vafaee M., Crews D. (2013). Epigenetic Control of Gonadal Aromatase (cyp19a1) in Temperature-Dependent Sex Determination of Red-Eared Slider Turtles. PLoS ONE.

[94] Parrott B.B., Kohno S., Cloy-McCoy J.A., Guillette L.J. (2014). Differential incubation temperatures result in dimorphic DNA methylation patterning of the SOX9 and aromatase promoters in gonads of alligator (*Alligator mississippiensis*) embryos. Biol. Reprod..

[95] Szyf M., Weaver I., Meaney M. (2007). Maternal care, the epigenome and phenotypic differences in behavior. Reprod. Toxicol..

[96] Danchin E., Pocheville A., Rey O., Pujol B., Blanchet S. (2019). Epigenetically facilitated mutational assimilation: Epigenetics as a hub within the inclusive evolutionary synthesis. Biol. Rev..

[97] Jablonka E.V.A., Raz G.A.L. (2009). Transgenerational epigenetic inheritance: Prevalence, mechanisms, and implications for the study of heredity and evolution. Q. Rev. Biol..

[98] Wang Y., Liu H., Sun Z. (2017). Lamarck rises from his grave: Parental environment-induced epigenetic inheritance in model organisms and humans. Biol. Rev..

[99] Quadrana L., Colot V. (2016). Plant Transgenerational Epigenetics. Annu. Rev. Genet..

[100] Morgan H.D., Santos F., Green K., Dean W., Reik W. (2005). Epigenetic reprogramming in mammals. Hum. Mol. Genet..

[101] Lee H.J., Hore T.A., Reik W. (2014). Reprogramming the methylome: Erasing memory and creating diversity. Cell Stem Cell.

[102] Daxinger L., Whitelaw E. (2012). Understanding transgenerational epigenetic inheritance via the gametes in mammals. Nat. Rev. Genet..

[103] Heard E., Martienssen R.A. (2014). Transgenerational epigenetic inheritance: Myths and mechanisms. Cell.

[104] Dias B.G., Ressler K.J. (2014). Parental olfactory experience influences behavior and neural structure in subsequent generations. Nat. Neurosci..

[105] Weyrich A., Benz S., Karl S., Jeschek M., Jewgenow K., Fickel J. (2016). Paternal heat exposure causes DNA methylation and gene expression changes of Stat3 in Wild guinea pig sons. Ecol. Evol..

[106] Nätt D., Rubin C.J., Wright D., Johnsson M., Beltéky J., Andersson L., Jensen P. (2012). Heritable genome-wide variation of gene expression and promoter methylation between wild and domesticated chickens. BMC Genom..

[107] Hu J., Wuitchik S.J.S., Barry T.N., Jamniczky H.A., Rogers S.M., Barrett R.D.H. (2021). Heritability of DNA methylation in threespine stickleback (*Gasterosteus aculeatus*). Genetics.

[108] Heckwolf M.J., Meyer B.S., Häsler R., Höppner M.P., Eizaguirre C., Reusch T.B.H. (2020). Two different epigenetic information channels in wild three-spined sticklebacks are involved in salinity adaptation. Sci. Adv..

[109] Walser J.C., Furano A. (2010). V The mutational spectrum of non-CpG DNA varies with CpG content. Genome Res..

[110] Hwang D.G., Green P. (2004). Bayesian Markov chain Monte Carlo sequence analysis reveals varying neutral substitution patterns in mammalian evolution. Proc. Natl. Acad. Sci. USA.

[111] Bird A.P. (1980). DNA methylation and the frequency of CpG in animal DNA. Nucleic Acids Res..

[112] Gorelick R. (2003). Evolution of dioecy and sex chromosomes via methylation driving Muller’s ratchet. Biol. J. Linn. Soc..

[113] Panchin A.Y., Makeev V.J., Medvedeva Y.A. (2016). Preservation of methylated CpG dinucleotides in human CpG islands. Biol. Direct.

[114] Kong A., Frigge M.L., Masson G., Besenbacher S., Sulem P., Magnusson G., Gudjonsson S.A., Sigurdsson A., Jonasdottir A., Jonasdottir A. (2012). Rate of de novo mutations and the importance of father’s age to disease risk. Nature.

[115] Amster G., Sella G. (2016). Life history effects on the molecular clock of autosomes and sex chromosomes. Proc. Natl. Acad. Sci. USA.

[116] Schmutte C., Yang A.S., Beart R.W., Jones P.A. (1995). Base Excision Repair of U:G Mismatches at a Mutational Hotspot in the p53 Gene Is More Efficient Than Base Excision Repair of T:G Mismatches in Extracts of Human Colon Tumors. Cancer Res..

[117] Bellacosa A., Drohat A.C. (2015). Role of base excision repair in maintaining the genetic and epigenetic integrity of CpG sites. DNA Repair.

[118] Gao Z., Wyman M.J., Sella G., Przeworski M. (2016). Interpreting the Dependence of Mutation Rates on Age and Time. PLoS Biol..

[119] MacRae S.L., Croken M.M.K., Calder R.B., Aliper A., Milholland B., White R.R., Zhavoronkov A., Gladyshev V.N., Seluanov A., Gorbunova V. (2015). DNA repair in species with extreme lifespan differences. Aging.

[120] Pfeifer G.P. (2006). Mutagenesis at methylated CpG sequences. Curr. Top. Microbiol. Immunol..

[121] Poulos R.C., Olivier J., Wong J.W.H. (2017). The interaction between cytosine methylation and processes of DNA replication and repair shape the mutational landscape of cancer genomes. Nucleic Acids Res..

[122] Shen J.C., Rideout W.M., Jones P.A. (1992). High frequency mutagenesis by a DNA methyltransferase. Cell.

[123] Tomkova M., McClellan M., Kriaucionis S., Schuster-Böckler B. (2018). DNA Replication and associated repair pathways are involved in the mutagenesis of methylated cytosine. DNA Repair.

[124] Ollila J., Lappalainen I., Vihinen M. (1996). Sequence specificity in CpG mutation hotspots. FEBS Lett..

[125] Branciamore S., Chen Z.-X., Riggs A.D., Rodin S.N. (2010). CpG island clusters and pro-epigenetic selection for CpGs in protein-coding exons of HOX and other transcription factors. Proc. Natl. Acad. Sci. USA.

[126] Kim S.H., Elango N., Warden C., Vigoda E., Yi S.V. (2006). Heterogeneous genomic molecular clocks in primates. PLoS Genet..

[127] Moorjani P., Amorim C.E.G., Arndt P.F., Przeworski M. (2016). Variation in the molecular clock of primates. Proc. Natl. Acad. Sci. USA.

[128] Alexandrov L.B., Nik-Zainal S., Wedge D.C., Aparicio S.A.J.R., Behjati S., Biankin A.V., Bignell G.R., Bolli N., Borg A., Børresen-Dale A.L. (2013). Signatures of mutational processes in human cancer. Nature.

[129] Gao Z., Moorjani P., Sasani T.A., Pedersen B.S., Quinlan A.R., Jorde L.B., Amster G., Przeworski M. (2019). Overlooked roles of DNA damage and maternal age in generating human germline mutations. Proc. Natl. Acad. Sci. USA.

[130] Harris R.S. (2013). Cancer mutation signatures, DNA damage mechanisms, and potential clinical implications. Genome Med..

[131] Qu J., Hodges E., Molaro A., Gagneux P., Dean M.D., Hannon G.J., Smith A.D. (2018). Evolutionary expansion of DNA hypomethylation in the mammalian germline genome. Genome Res..

[132] Saxonov S., Berg P., Brutlag D.L. (2006). A genome-wide analysis of CpG dinucleotides in the human genome distinguishes two distinct classes of promoters. Proc. Natl. Acad. Sci. USA.

[133] Fryxell K.J., Moon W.J. (2005). CpG mutation rates in the human genome are highly dependent on local GC content. Mol. Biol. Evol..

[134] Molaro A., Hodges E., Fang F., Song Q., McCombie W.R., Hannon G.J., Smith A.D. (2011). Sperm methylation profiles reveal features of epigenetic inheritance and evolution in primates. Cell.

[135] Xia J., Han L., Zhao Z. (2012). Investigating the relationship of DNA methylation with mutation rate and allele frequency in the human genome. BMC Genom..

[136] Zhi D., Aslibekyan S., Irvin M.R., Claas S.A., Borecki I.B., Ordovas J.M., Absher D.M., Arnett D.K. (2013). SNPs located at CpG sites modulate genome-epigenome interaction. Epigenetics.

[137] Hanson H.E., Wang C., Schrey A.W., Liebl A.L., Ravinet M., Jiang R.H.Y., Martin L.B. (2022). Epigenetic Potential and DNA Methylation in an Ongoing House Sparrow *(Passer domesticus)* Range Expansion. Am. Nat..

[138] Shi Y., Xu L., Feng Q., Li A., Jia J., Xu Y., Yang D., Zhang Y., Zhang X., Zhao H. (2018). Allele-specific methylation contributed by CpG-SNP is associated with regulation of ALOX5AP gene expression in ischemic stroke. Neurol. Sci..

[139] Izzi B., Pistoni M., Cludts K., Akkor P., Lambrechts D., Verfaillie C., Verhamme P., Freson K., Hoylaerts M.F. (2016). Allele-specific DNA methylation reinforces PEAR1 enhancer activity. Blood.

[140] Shoemaker R., Deng J., Wang W., Zhang K. (2010). Allele-specific methylation is prevalent and is contributed by CpG-SNPs in the human genome. Genome Res..

[141] Wang H., Lou D., Wang Z. (2019). Crosstalk of genetic variants, allele-specific DNA methylation, and environmental factors for complex disease risk. Front. Genet..

[142] Hanson H.E., Zimmer C., Koussayer B., Schrey A.W., Maddox J.D., Martin L.B. (2021). Epigenetic Potential Affects Immune Gene Expression in House Sparrows. J. Exp. Biol..

[143] Agrawal A.A. (2020). A scale-dependent framework for trade-offs, syndromes, and specialization in organismal biology. Ecology.

[144] Lande R. (2015). Evolution of phenotypic plasticity in colonizing species. Mol. Ecol..

[145] Hanson H.E., Koussayer B., Kilvitis H.J., Schrey A.W., Maddox J.D., Martin L.B. (2020). Epigenetic Potential in Native and Introduced Populations of House Sparrows (*Passer domesticus*). Integr. Comp. Biol..

[146] Liebl A.L., Schrey A.W., Richards C.L., Martin L.B. (2013). Patterns of DNA methylation throughout a range expansion of an introduced songbird. Integr. Comp. Biol..

[147] Schrey A.W., Coon C.A.C., Grispo M.T., Awad M., Imboma T., McCoy E.D., Mushinsky H.R., Richards C.L., Martin L.B. (2012). Epigenetic Variation May Compensate for Decreased Genetic Variation with Introductions: A Case Study Using House Sparrows (*Passer domesticus*) on Two Continents. Genet. Res. Int..

[148] Martin L.B., Liebl A.L. (2014). Physiological flexibility in an avian range expansion. Gen. Comp. Endocrinol..

[149] Liebl A.L., Martin L.B. (2012). Exploratory behaviour and stressor hyper responsiveness facilitate range expansion of an introduced songbird. Proc. R. Soc. B Biol. Sci..

[150] Liebl A.L., Martin L.B. (2014). Living on the edge: Range edge birds consume novel foods sooner than established ones. Behav. Ecol..

[151] Storz J.F., Natarajan C., Signore A.V., Witt C.C., McCandlish D.M., Stoltzfus A. (2019). The role of mutation bias in adaptive molecular evolution: Insights from convergent changes in protein function. Philos. Trans. R. Soc. B Biol. Sci..

[152] Guerrero-Bosagna C. (2019). From epigenotype to new genotypes: Relevance of epigenetic mechanisms in the emergence of genomic evolutionary novelty. Semin. Cell Dev. Biol..

[153] Guerrero-Bosagna C. (2012). Finalism in Darwinian and Lamarckian Evolution: Lessons from Epigenetics and Developmental Biology. Evol. Biol..

[154] Guerrero-Bosagna C., Sabat P., Valladares L. (2005). Environmental signaling and evolutionary change: Can exposure of pregnant mammals to environmental estrogens lead to epigenetically induced evolutionary changes in embryos?. Evol. Dev..

[155] Flores K.B., Wolschin F., Amdam G.V. (2013). The role of methylation of DNA in environmental adaptation. Integr. Comp. Biol..

[156] Bateson P., Gluckman P. (2011). Plasticity, Robustness, Development and Evolution.

[157] Flores K.B., Amdam G.V. (2011). Deciphering a methylome: What can we read into patterns of DNA methylation?. J. Exp. Biol..

[158] Mugal C.F., Arndt P.F., Holm L., Ellegren H. (2015). Evolutionary consequences of DNA methylation on the GC content in vertebrate genomes. G3 Genes Genomes Genet..

[159] Simmen M.W. (2008). Genome-scale relationships between cytosine methylation and dinucleotide abundances in animals. Genomics.

[160] Zhou Y.H., Zheng J.B., Gu X., Saunders G.F., Alfred Yung W.K. (2002). Novel PAX6 binding sites in the human genome and the role of repetitive elements in the evolution of gene regulation. Genome Res..

[161] Zemojtel T., Kiebasa S.M., Arndt P.F., Behrens S., Bourque G., Vingron M. (2011). CpG deamination creates transcription factor-binding sites with high efficiency. Genome Biol. Evol..

[162] Zemojtel T., Kielbasa S.M., Arndt P.F., Chung H.R., Vingron M. (2009). Methylation and deamination of CpGs generate p53-binding sites on a genomic scale. Trends Genet..

[163] He X., Tillo D., Vierstra J., Syed K.S., Deng C., Ray G.J., Stamatoyannopoulos J., FitzGerald P.C., Vinson C. (2015). Methylated Cytosines Mutate to Transcription Factor Binding Sites that Drive Tetrapod Evolution. Genome Biol. Evol..

[164] Galen S.C., Natarajan C., Moriyama H., Weber R.E., Fago A., Benham P.M., Chavez A.N., Cheviron Z.A., Storz J.F., Witt C.C. (2015). Contribution of a mutational hot spot to hemoglobin adaptation in high-Altitude Andean house wrens. Proc. Natl. Acad. Sci. USA.

[165] Ying H., Huttley G. (2011). Exploiting CpG hypermutability to identify phenotypically significant variation within human protein-coding genes. Genome Biol. Evol..

[166] Mikkelsen T.S., Hillier L.W., Eichler E.E., Zody M.C., Jaffe D.B., Yang S.P., Enard W., Hellmann I., Lindblad-Toh K., Altheide T.K. (2005). Initial sequence of the chimpanzee genome and comparison with the human genome. Nature.

[167] Pértille F., Da Silva V.H., Johansson A.M., Lindström T., Wright D., Coutinho L.L., Jensen P., Guerrero-Bosagna C. (2019). Mutation dynamics of CpG dinucleotides during a recent event of vertebrate diversification. Epigenetics.

[168] Cooper D.N., Youssoufian H. (1988). The CpG dinucleotide and human genetic disease. Hum. Genet..

[169] Cooper D.N., Mort M., Stenson P.D., Ball E.V., Chuzhanova N.A. (2010). Methylation-mediated deamination of 5-methylcytosine appears to give rise to mutations causing human inherited disease in CpNpG trinucleotides, as well as in CpG dinucleotides. Hum. Genomics.

[170] Chae H., Lee S., Nephew K.P., Kim S. (2016). Subtype-specific CpG island shore methylation and mutation patterns in 30 breast cancer cell lines. BMC Syst. Biol..

